# Role of Clofazimine in Treatment of *Mycobacterium avium* Complex

**DOI:** 10.3389/fmed.2021.638306

**Published:** 2021-04-15

**Authors:** Mohammad Javad Nasiri, Tess Calcagno, Sareh Sadat Hosseini, Ali Hematian, Neda Yousefi Nojookambari, Mohammadmahdi Karimi-Yazdi, Mehdi Mirsaeidi

**Affiliations:** ^1^Department of Microbiology, School of Medicine, Shahid Beheshti University of Medical Sciences, Tehran, Iran; ^2^Department of Medicine, University of Miami, Miami, FL, United States; ^3^Student Research Committee, School of Medicine, Shahid Beheshti University of Medical Sciences, Tehran, Iran; ^4^Faculty of Paramedical Sciences, Mazandaran University of Medical Sciences, Sari, Iran; ^5^Division of Pulmonary and Critical Care, University of Miami, Miami, FL, United States

**Keywords:** clofazimine, *Mycobacterium avium* complex, pulmonary disease, mycobacteria, MAC

## Abstract

**Background:** Non-tuberculous mycobacteria (NTM), specifically *Mycobacterium avium* complex (MAC), is an increasingly prevalent cause of pulmonary dysfunction. Clofazimine has been shown to be effective for the treatment of *M. avium* complex, but there were no published large-scale analyses comparing clofazimine to non-clofazimine regimens in MAC treatment. The objective of this large-scale meta-analysis was to evaluate patient characteristics and treatment outcomes of individuals diagnosed with MAC and treated with a clofazimine-based regimen.

**Methods:** We used Pubmed/Medline, Embase, Web of Science, and the Cochrane Library to search for studies published from January 1, 1990 to February 9, 2020. Two reviewers (SSH and NY) extracted the data from all eligible studies and differences were resolved by consensus. Statistical analyses were performed with STATA (version 14, IC; Stata Corporation, College Station, TX, USA).

**Results:** The pooled success treatment rate with 95% confidence intervals (CI) was assessed using random effect model. The estimated pooled treatment success rates were 56.8% in clofazimine and 67.9% in non-clofazimine groups. Notably, success rates were higher (58.7%) in treatment of HIV patients with disseminated infection.

**Conclusions:** Treatment was more successful in the non-clofazimine group overall. However, HIV patients with disseminated infection had higher treatment response rates than non-HIV patients within the clofazimine group.

## Introduction

### Non-tuberculous Mycobacteria

Non-tuberculous mycobacteria (NTM) are found ubiquitously in the environment and serve as a common cause of pulmonary infection associated with increasing prevalence and significantly impaired health-related quality of life (HRQL). Symptoms of pulmonary NTM (PNTM) are non-specific (cough, fever, malaise) and severity is dependent on presence of baseline lung comorbidities (1). The majority (80%) of PNTM infections are caused by *Myobacterium avium* complex (MAC) (2–5).

### Current Treatment Approaches

There are limited data to guide the treatment of pulmonary non-tuberculous mycobacterial infection in patients without HIV. Current strategies involve multimodal drug therapy, drug susceptibility testing, and extended courses of antimicrobials which can often be unsuccessful. Extended courses of targeted drug therapy for slow verses rapid growing NTM are selected with the assistance of drug susceptibility testing (DST) (4). The American Thoracic Society/Infectious Diseases Society of America guidelines recommend a three-drug macrolide combination therapy containing rifamycin (rifampin, rifapentine, or rifabutin), ethambutol, with a macrolide (clarithromycin or azithromycin) for at least 12 months after culture conversion. The addition of aminoglycoside therapy (amikacin or streptomycin) is recommended in the first 3–6 months of therapy for severe disease (6).

### Role of Clofazimine

Continued discovery is crucial to streamline a treatment regimen for PNTM in attempt to lower costs, target treatment-resistant isolates, and increase health-related quality of life for patients. Clofazimine is a lipophilic antibiotic FDA approved for the treatment of *Mycobacterium leprae*, the bacteria causing Hansen's disease. Clofazimine inhibits mycobacterial respiratory chain and ion transporters in the outer membrane; the phenazine molecule acts as an artificial electron acceptor. Clofazimine is oxidized in place of NADH, leading to reduced cellular ATP and presence of damaging reactive oxygen species (7). The role of clofazimine in the treatment of MAC has not been elucidated. Its efficacy has been shown in several studies, but a comprehensive analysis has not been published. The objective of this large-scale meta-analysis was to evaluate patient characteristics and treatment outcomes of individuals diagnosed with MAC who were treated with a clofazimine based regimen.

## Experimental Section

### Search Strategy

We searched Pubmed/Medline, Embase, Web of science and the Cochrane Library for studies published from January 1, 1990 to February 9, 2020. The search strategy was based on the following key words: *M. avium* complex, *Mycobacterium avium-intracellulare* complex, MAC, macrolides, aminoglycosides, and clofazimine. Lists of references of selected articles and relevant review articles were hand-searched to identify further studies. Only studies written in English were selected. This study was conducted and reported according to the PRISMA guidelines (8).

### Study Selection

The records found through database searching were merged and the duplicates were removed using EndNote X7 (Thomson Reuters, New York, NY, USA). Two reviewers (SSH and NY) independently screened the records by title and abstract to exclude those not related to the current study. The full-text of potentially eligible records was retrieved and evaluated by a third reviewer (MJN). Included studies met the following inclusion criteria: (i) patients were diagnosed with MAC using the criteria suggested by ATS/ IDSA; (ii) all study patients were treated with clofazimine or macrolide and/or aminoglycoside-containing regimens, with companion drugs; and (iii) the treatment outcomes were addressed. We defined treatment success as achievement of culture conversion and completion of the planned treatment without relapse while on treatment. Studies with insufficient information about patients' characteristics and treatment outcomes were excluded. Conference abstracts, editorials, and reviews were also excluded.

### Data Extraction and Quality Assessment

A data extraction form was designed by two reviewers (SSH and NY). These reviewers extracted the data from all eligible studies and differences were resolved by consensus. The following data were extracted: first author name; year of publication; study duration, type of study, country/ies where the study was conducted; number of patients with MAC; age; HIV/AIDS status; treatment protocols (treatment regimens and duration of treatment), and treatment outcome. The methodological quality of the eligible studies was assessed according to the Cochrane-based criteria (9).

### Data Synthesis and Analysis

Statistical analyses were performed with STATA (version 14, IC; Stata Corporation, College Station, TX, USA). The pooled success treatment rate with 95% confidence intervals (CI) was assessed using random effect model. The between-study heterogeneity was assessed by Cochran's Q and the *I*2 statistic. Publication bias was assessed statistically by using Begg's and Egger’s-tests (*p* < 0.05) was considered indicative of statistically significant publication bias). To explore sources of studies’ heterogeneity, sensitivity analyses were carried out with meta-regression and subgroup analysis.

## Results

**Figure 1:**
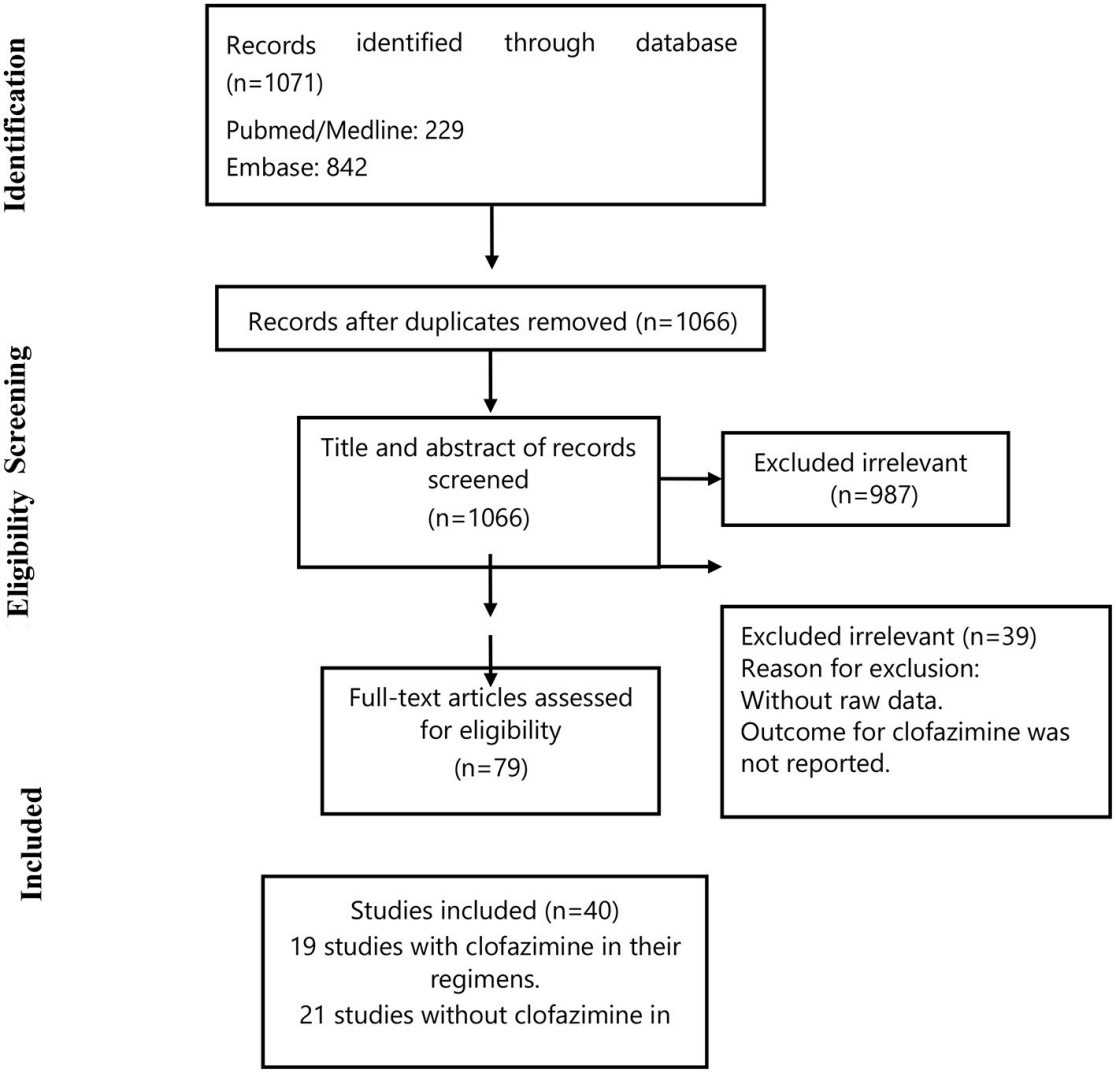
Flow chart of study selection for inclusion in the systematic review and meta-analysis.

Figure 1 summarizes the study selection process. Briefly, we retrieved data from 40 selected articles comprising data for 19 studies with clofazimine in their regimens (clofazimine group) and 21 studies without clofazimine in their regimens (Non-clofazimine group). Characteristics of the included studies are described in Tables 1, 2.

**Table 1:** Characteristics of studies with clofazimine in their regimens.

**References**	**Country**	**Type of study**	**HIV prevalence (%)**	**Mean age**	**MAC disease**	**Sample size**	**Treatment regimens**	**Median length of treatment (months)**	**Definition of cure**
Aznar et al. (10)	Canada	Retrospective	NR	61	MAC pulmonary disease	35	CFZ+RFP+EMB+AMK+FQ+macrolide	26	Culture conversion
									Symptom improvement
Martiniano et al. (11)	USA	Prospective cohort	0	67	MAC pulmonary disease	26	CFZ+RFP+EMB+AMK+FQ+macrolide	12	Culture conversion
Jarand et al. (12)	Canada	Retrospective	0	67	MAC pulmonary disease	107	CFZ+EMB+macrolide	14	Culture conversion
Jo et al. (13)	South Korea	Retrospective	0	59	MAC pulmonary disease	51	CFZ+MXF+RFB	5	Culture conversion
Field and Cowie (14)	Canada	NR	0	70	MAC pulmonary disease	30	CFZ+CLR+AZM+EMB	12	Culture conversion
Singer et al. (15)	Canada	Randomized trial	100	16≤	Disseminate d MAC disease	90	CFZ+RFP+EMB+CPX	4	Symptom improvement
Cohn et al. (16)	USA	Randomized trial	100	38	Disseminate d MAC disease	28	CFZ or RFB+CLR 500 mg+EMB	2	Culture conversion
						26	CFZ or RFB+CLR 1,000 mg+EMB	2	Culture conversion
Fournier et al. (17)	France	Randomized trial	100	39	Disseminate d MAC disease	16	CFZ+CLR+EMB	2	Culture conversion
Haefner et al. (18)	Switzerland	Randomized trial	100	40	Disseminate d MAC disease	23	CFZ+CLR+RFB	4.5	Culture conversion
									Symptom improvement
Burman et al. (19)	USA	Retrospective cohort	100	35	Disseminate d MAC disease	117	CFZ+CLR+EMB	3	Symptom improvement
Parenti et al. (20)	USA	Randomized trial	100	36	Disseminate d MAC disease	37	CFZ+RFP+CPX+EMB+AMK	3	Culture conversion
							CFZ+RFP+CPX+EMB	3	Culture conversion
Roussel and Igual (21)	France	NR	0	41	MAC pulmonary disease	22	CFZ+CLR+Mino	15	Culture conversion
Chaisson et al. (22)	USA	Randomized trial	100	37	Disseminate d MAC disease	51	CFZ+CLR+EMB	3	Culture conversion
Dube et al. (23)	USA	Randomized trial	100	37	Disseminate d MAC disease	21	CFZ+CLR	2	Culture conversion
						31	CFZ+CLR+EMB	2	Culture conversion
May et al. (24)	France	Randomized trial	100	35	Disseminate d MAC disease	59	CFZ+CLR	2	Culture conversion
Shafran et al. (25)	Canada	Randomized trial	100	38	Disseminate d MAC disease	90	CFZ+RFP+EMB+CPX	3	Culture conversion
Dautzenberg et al. (26)	France	Randomized trial	100	37	Disseminate d MAC disease	55	CFZ+RFB+EMB+INH	3	Culture conversion
						47	CFZ+EMB+INH	3	Culture conversion
Kissinger et al. (27)	USA	Randomized trial	100	33	Disseminate d MAC disease	29	CFZ+EMB+CPX+RFP	3	Symptom improvement
						44	CFZ+EMB+CPX+RFP+CLR	3	Symptom improvement
Kemper et al. (28)	USA	Randomized trial	100	35	Disseminate d MAC disease	31	RFP+EMB+CFZ+CPX+AMK	3	Culture conversion

*EMB, etambutol; RFP, Rifampicin; RFB, Rifabutin; INH, isoniazid; STM, streptomycin; CFZ, clofazimine; CPX, ciprofloxacin; CLR, clarithromycin; AZM, azithromycin; AMK, amikacin; Mino, minocycline; FQ, fluoroquinolone*.

**Table 2:** Characteristics of studies without clofazimine in their regimens.

**References**	**Country**	**Type of study**	**HIV prevalence (%)**	**Mean age**	**MAC disease**	**Sample size**	**Treatment regimens**	**Median length of treatment (months)**	**Definition of cure**
Asakura et al. (29)	Japan	Retrospectiv e	0	68	Refractory MAC pulmonary disease	31	STFX+CLR+EMB+RFP	12	Culture conversion
									Radiologic improvement
									Symptom improvement
Jhun et al. (30)	South Korea	Prospective cohort	NR	63	MAC pulmonary disease	26	EMB+RFP+macrolide	23.2	Culture conversion
									Radiologic improvement
									Symptom improvement
Cadelis et al. (31)	France	Retrospectiv e	17	50	MAC pulmonary disease	34	CLR+RFP+EMB	8.4	Culture conversion
Zweijpfenning et al. (32)	Netherland s	Retrospectiv e	NR	61	MAC pulmonary disease	34	RFP+EMB+macrolide	15.7	Culture conversion
									Radiologic improvement
									Symptom improvement
Ellender et al. (33)	Australia	Retrospectiv e cohort	NR	61	MAC pulmonary disease	31	CLR+RFP+EMB+AMK	NR	Culture conversion
									Symptom improvement
Griffith et al. (34)	USA	Retrospectiv e	NR	75	MAC pulmonary disease	180	CLR+RFP+EMB	>12	Culture conversion
Shimomura et al. (35)	Japan	Retrospectiv e cohort	NR	71	MAC pulmonary disease	42	CLR+RFP+EMB	12	Culture conversion
Ito et al. (36)	Japan	Retrospectiv e	0	61	MAC pulmonary disease	72	CLR+RFP+EMB	>12	Culture conversion
Miwa et al. (37)	Japan	Randomized trial	0	68	MAC pulmonary disease	32	CLR+RFP+EMB	12	Culture conversion
Fujita et al. (38)	Japan	Randomized trial	0	69	MAC pulmonary disease	14	CLR+RFP+EMB	12	Culture conversion
									Radiologic improvement
									Symptom improvement
Kim et al. (39)	South Korea	Retrospectiv e	NR	65	MAC pulmonary disease	21	CLR+RFP+EMB	18	Culture conversion
									Radiologic improvement
									Symptom improvement
Sim et al. (40)	South Korea	Retrospectiv e	0	59	MAC pulmonary disease	96	CLR+RFP+EMB	>12	Culture conversion
									Radiologic improvement
									Symptom improvement
Hasegawa et al. (41)	Japan	Retrospectiv e	NR	62	MAC pulmonary disease	13	CLR+RFP+EMB	18	Culture conversion
Jenkins et al. (42)	UK	Randomized trial	0	67	MAC pulmonary disease	66	CLR+EMB+RFB	24	Culture conversion
Kobashi et al. (43)	Japan	Randomized trial	0	63	MAC pulmonary disease	73	CLR+ RFB+EMB	24	Culture conversion
									Symptom improvement
Lam et al. (44)	USA	Randomized trial	0	60	MAC pulmonary disease	91	CLR+RFP/RFB+EMB	>12	Culture conversion
									Radiologic improvement
									Symptom improvement
Benson et al. (45)	USA	Randomized trial	100	35	Disseminat ed MAC disease	57	CLR+RFB+EMB	16 week	Culture conversion
Dunne et al. (46)	USA	Randomized trial	100	36	Disseminat ed MAC disease	57	CLR+EMB	6	Culture conversion
Gordin et al. (47)	USA	Randomized trial	100	36	Disseminat ed MAC disease	70	CLR+EMB+RFB	4	Culture conversion
Tanaka et al. (48)	Japan	NR	0	60	MAC pulmonary disease	39	CLR+EMB+ RFB+KAN+OFX or LVX	>6	Culture conversion
Wallace et al. (49)	USA	NR	0	60	MAC pulmonary disease	39	CLR+EMB+RFP	6	Culture conversion

*STFX, sitafloxacin; EMB, etambutol; RFP, Rifampicin; RFB, Rifabutin; INH, isoniazid; STM, streptomycin; CLR, clarithromycin; AMK, amikacin; OFX, ofloxacin; LVX, levofloxacin; KAN, kanamycin*.

### Quality Assessment

Based on Cochrane tool (Table 3), the included studies had a low risk of bias. In clofazimine group, 12 studies were randomized controlled trials and the rest were non-randomized controlled trials (i.e., cohort or retrospective observational studies). In this group, the statistical analysis methodology was well-described in 17 studies but was not reported in the other two studies.

**Table 3:** Assessment of study quality.

**Studies**	**First author**	**Sampling methods**	**Blinded**	**Cross sectional design**	**Prospective**	**Incomplete outcome data addressed**
Studies with clofazimine in their regimens	Aznar	Consecutive	No	Yes	No	No
	Martiniano	Consecutive	No	Yes	No	No
	Jarand	Consecutive	No	Yes	No	No
	Jo	Consecutive	No	Yes	No	No
	Field	Consecutive	No	Yes	No	No
	Singer	Randomized	No	Yes	Yes	No
	Cohn	Randomized	No	Yes	Yes	Yes
	Fournier	Randomized	No	Yes	Yes	No
	Haefner	Consecutive	NR	Yes	Yes	No
	Burman	Consecutive	No	Yes	No	No
	Parenti	Randomized	NR	Yes	Yes	No
	Roussel	Consecutive	No	Yes	Yes	Yes
	Chaisson	Randomized	No	Yes	Yes	No
	Dube	Randomized	No	Yes	Yes	No
	May	Randomized	No	Yes	Yes	No
	Shafran	Randomized	No	Yes	Yes	No
	Dautzenberg	Randomized	Yes	Yes	Yes	No
	Kissinger	Randomized	No	Yes	Yes	No
	Kemper	Randomized	No	Yes	Yes	No
Studi	Asakura	Consecutive	No	Yes	No	No
	Jhun	Consecutive	No	Yes	Yes	No
	Cadelis	Consecutive	No	Yes	No	No
	Zweijpfenning	Consecutive	No	Yes	No	No
	Ellender	Consecutive	No	Yes	No	No
	Griffith	Consecutive	No	Yes	No	No
	Shimomura	Consecutive	No	Yes	No	No
	Ito	Consecutive	No	Yes	No	No
	Miwa	Randomized	No	Yes	Yes	No
	Fujita	Randomized	No	Yes	Yes	No
	Kim	Consecutive	No	Yes	No	No
	Sim	Consecutive	No	Yes	No	No
	Hasegawa	Consecutive	No	Yes	No	No
	Jenkins	Randomized	No	Yes	Yes	Yes
	Kobashi	Randomized	Yes	Yes	Yes	No
	Lam	Randomized	Yes	Yes	Yes	No
	Benson	Randomized	No	Yes	Yes	No
	Dunne	Randomized	Yes	Yes	Yes	Yes
	Gordin	Randomized	No	Yes	Yes	No
	Tanaka	Consecutive	No	No	No	No
	Wallace	Consecutive	No	Yes	No	No

### Treatment Success

**Figure 2:**
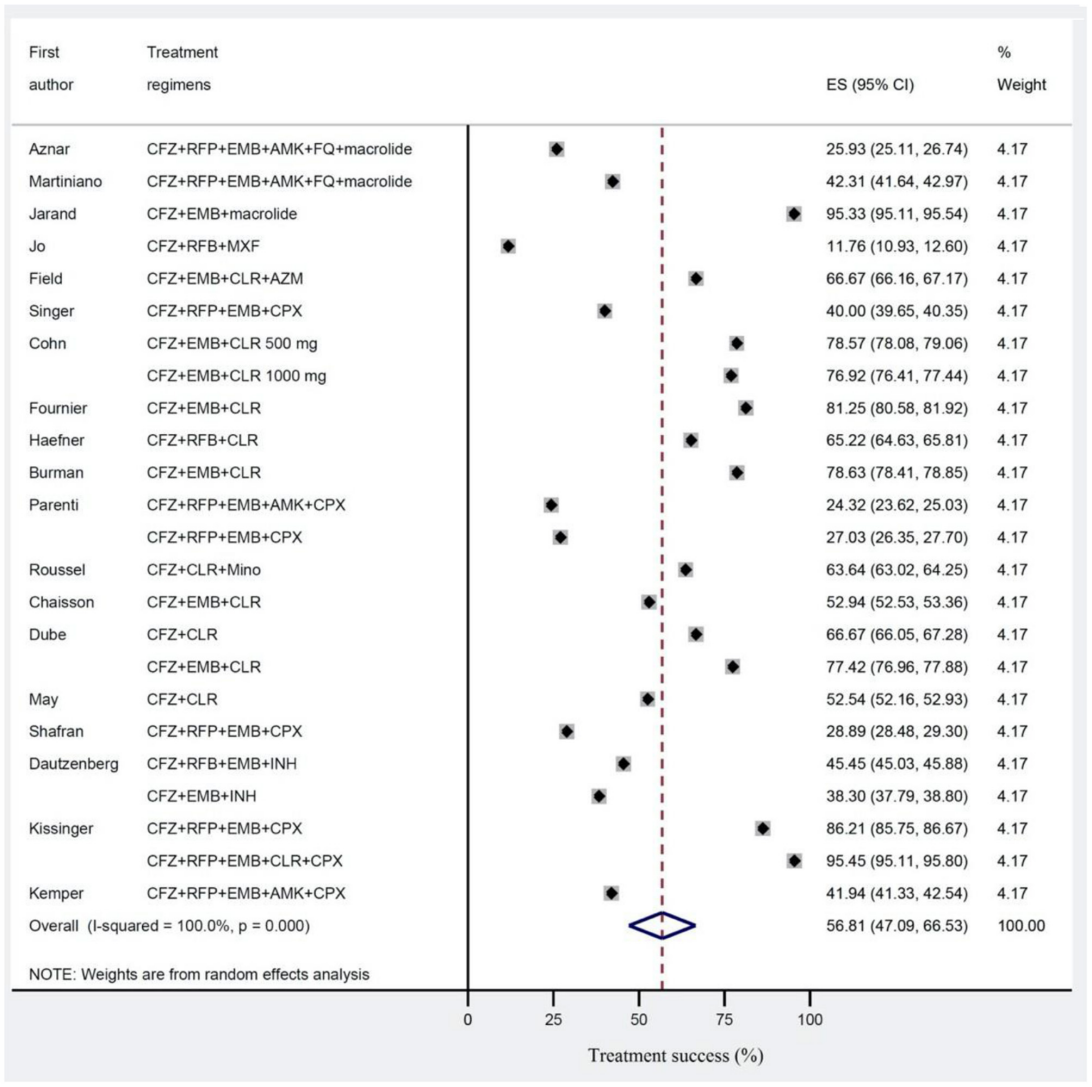
Treatment success for *Mycobacterium avium* complex (MAC) disease in studies with clofazimine in their regimens. Treatment effects and summaries were calculated using a random-effects model weighted by study population.

**Figure 3:**
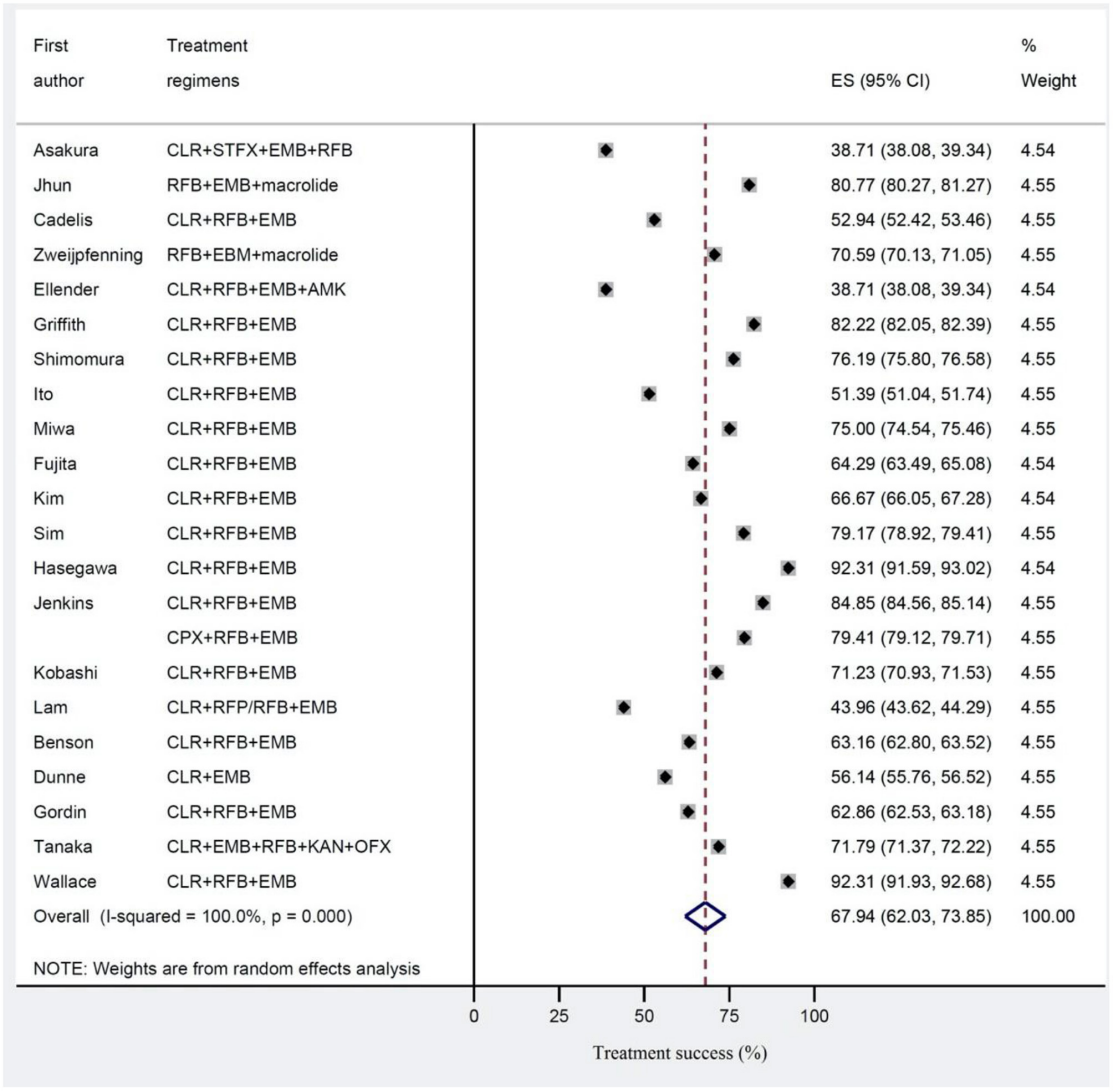
Treatment success for *Mycobacterium avium* complex (MAC) disease in studies without clofazimine in their regimen.

The estimated pooled treatment success rates were found to be 56.8% (95% CI 47.0–66.5%) and 67.9% (95% CI 62.0–73.8%) in clofazimine and non-clofazimine groups, respectively (Figures 2, 3). The heterogeneity in the study characteristics led to significant variation in the reported treatment outcomes. Varying treatment success rates caused heterogeneity in the pooled results. Thus, we ran a meta-regression to understand the source of heterogeneity. Based on meta-regression, different treatment success rates resulted as a significant source of heterogeneity (*P*-value = 0.000) in both clofazimine and non-clofazimine groups. In clofazimine group, there was some evidence of publication bias (Begg’s and tests *P*-value was 0.01).

### Subgroup Analysis

Table 4 shows the subgroup analysis of the studies based on treatment regimens, length of treatment, type of patients, number of drugs used, definition of cure, type of study and year of publication.

**Table 4:** Pooled treatment success among subgroups of studies with clofazimine in their regimens.

**Subgroups**	**No. of study**	**Treatment success (95% CI)**	**Heterogeneity**	
			***p*-value**	***I*2 (%)**
**Treatment regimens**				
Clofazimine-containing regimens	19 studies	56.8 (47.0–66.5)	0.000	100
Non-clofazimine containing regimens	21 studies	67.9 (62.0–73.8)	0.000	100
**Length of treatment**				
≥12 Months	5 studies	58.7 (33.1–84.3)	0.000	100
<12 Months	14 studies	56.2 (46.0–66.6)	0.000	88
**Type of patients**				
Non-HIV patients with MAC pulmonary disease	6 studies	51.0 (24.1–77.7)	0.000	100
HIV patients with disseminated MAC disease	13 studies	58.7 (48.7-69.0)	0.000	100
**Number of drugs used**				
≤3	11 studies	64.5 (53.7–75.3)	0.000	100
>3	9 studies	47.6 (31.5–63.7)	0.000	100
**Definition of cure**				
Culture conversion	16 studies	53.1 (42.0–64.3)	0.000	100
Symptom improvements	5 studies	71.0 (53.1–88.7)	0.000	100
**Type of study**				
Randomized trials	12 studies	57.5 (46.6–68.4)	0.000	100
Non-randomized trials	7 studies	54.8 (37.3–72.4)	0.000	100
**Year of publication**				
>2,000	5 studies	47.4 (19.0–75.0)	0.000	100
≤2,000	14 studies	60.2 (50.6–69.7)	0.000	100

## Discussion

### Summary

This study found that the estimated pooled treatment success rates were 56.8% in clofazimine and 67.9% in non-clofazimine groups. The duration of treatment above 1 year after did not show any improvement in success rates. The success rate was higher (58.7%) in treatment of HIV patients with disseminated MAC compared to treatment of Non-HIV patients with MAC pulmonary disease (51.0%). Counterintuitively, treatment regimens containing more than three drugs were less successful (47.6% compared to 64.5%). The success rates were higher in studies which defined cure by symptomatic improvement rather than culture conversion.

### Clofazimine

The need for novel therapies to combat MAC infection is high due to drug resistance, disease recurrence, and current suboptimal efficacy. Even though there is a lack of robust data supporting its efficacy, clofazimine has been used in combination therapies for the treatment of MAC. In this study we found lower treatment success rates when using clofazimine-based regimens, especially for the treatment of non-HIV related MAC pulmonary disease.

Resistance to clofazimine may have contributed to lower treatment success rates. *In vitro* isolates of NTM have been shown to be susceptible to clofazimine. Luo et al. tested 209 isolates containing rapid and slow growing NTM for *in vitro* susceptibility to clofazimine. Most slow growing clinical isolates were sensitive to clofazimine with MICs <1 μg/ml and 17 out of 30 rapid growing clinical isolates showed sensitivity with MICs below <1 μg/ml (50). However, Chen et al. found mutations in genes coding for transcriptional regulatory proteins of Mycobacterium which confer resistance to clofazimine (51).

In addition to its antimicrobial activity, clofazimine displays immune modulating effects which may alter patient response to therapy. Clofazimine increases humoral immune response by increasing major histocompatibility complex class II expression in monocytes and decreasing suppressor T-cell activity. However, it negatively modulates innate immune activity by inducing apoptosis in macrophages (52). It is possible that the clofazimine decreases cure rates through death of macrophages, however this decrease may be offset by the beneficial T-cell modulation in immunocompromised HIV patients.

Lower treatment success rates in the clofazimine group could be attributed to a clinically significant drug interaction with rifampin. Pooled treatment success rates were lower in regimens containing more than three drugs compared to regimens containing three or less drugs. Interestingly, every clofazimine study using a greater than three-drug regimen contained rifampin as part its regimen. Whereas, rifampin was only used in a minority of drug regimens containing ≤3 drugs (2/11). Rifamycin antimicrobials induce many hepatic cytochrome P450 enzymes as well as glucuronidation pathways. Clofazimine undergoes glucuronidation prior to excretion as it transitions out of its pharmacologically active state. Clinically noticeable drug-interactions have been reported between clofazimine and rifampin (53). This finding could be secondary to rifampin-induced glucuronidation and subsequent excretion of clofazimine.

### Previous Data

Clofazimine based treatment regimens have been shown to be efficacious for the treatment on MAC in several previous studies, but data is scant, and treatments were never compared prospectively in a head-to-head fashion. Field et al. conducted a single-arm prospective study looking at the efficacy of macrolide/ethambutol/clofazimine regimen in MAC lung disease in 33 patients. Treatment for an average of 10 months converted sputum findings to negative in 87% of patients. However, relapse occurs in 19% of patients (14). Jarand et al. retrospectively reviewed patients with MAC lung disease being treated with regimens including clofazimine or rifampin and found a higher culture conversion rate in the clofazimine group rifampin (100 vs. 71%; *P* = 0.0002). However, relapse and re-treatment rates did not differ between groups (12). Martiniano et al. retrospectively found 50% of patients with pulmonary disease converted to negative cultures with the treatment of clofazimine regimens.

However, only 48% of patient had *M. avium* complex, 21% of patients were diagnosed with cystic fibrosis, and most patients (78%) had failed previous treatment attempts (11).

### Strengths/Limitations

This study was the first comprehensive review comparing clofazimine and non-clofazimine treatment regimens for the treatment of MAC. Our sample size of studies (19) and controls (21) is robust. External validity of this paper is strong; we were able to include studies treating HIV related disseminated MAC and non-HIV related pulmonary MAC. However, there are some limitations to address. Our study did not characterize adverse effects and treatment adherence to clofazimine regimens. Adherence and associated adverse effects may have contributed to outcomes. Also, based on meta-regression, different treatment success rates resulted as a significant source of heterogeneity (*P*-value = 0.000) in both clofazimine and non-clofazimine groups. In the clofazimine group, there was some evidence of publication bias (Begg’s and tests *P*-value was 0.01). Interestingly, publication later than 2,000 showed lower success rates overall which could be explained by publication bias. However, subgroup analysis was conducted for the clofazimine treated group to compare heterogenous of the studies features.

### Future Directions

Our findings will help in assigning a role to clofazimine in the treatment of MAC. Based on our results, clofazimine should be considered a last-line agent. It is possible that its only role is in the treated of disseminated HIV disease. Future clinical trials need to be done to assess the efficacy in disseminated MAC. Furthermore, we need novel therapeutic agents to target non-HIV pulmonary MAC as current therapies are long in duration and often result in relapse of disease process (54).

## Data Availability Statement

The raw data supporting the conclusions of this article will be made available by the authors, without undue reservation.

## Author Contributions

MN performed the literature review, conducted data analysis, and manuscript preparation. TC performed the literature review and manuscript preparation. SH, AH, NN, and MK-Y helped in the literature review and data analysis. MM conducted literature review, designed the study, and performed data analysis and manuscript preparation. All authors contributed to the article and approved the submitted version.

## Conflict of Interest

The authors declare that the research was conducted in the absence of any commercial or financial relationships that could be construed as a potential conflict of interest.
